# Low mtDNA diversity in a highly differentiated population of spinner dolphins (*Stenella longirostris*) from the Fernando de Noronha Archipelago, Brazil

**DOI:** 10.1371/journal.pone.0230660

**Published:** 2020-04-07

**Authors:** Drienne Messa Faria, José Martins da Silva, Leonora Pires Costa, Samuel Rezende Paiva, Celso Luis Marino, Mario Manoel Rollo, C. Scott Baker, Ana Paula Cazerta Farro

**Affiliations:** 1 Departamento de Ciências Biológicas, Universidade Federal do Espírito Santo (UFES), Vitória, Espírito Santo, Brazil; 2 Instituto Chico Mendes de Conservação da Biodiversidade (ICMBio), Fernando de Noronha, Pernambuco, Brazil; 3 Departamento de Ciências Biológicas, Universidade Federal do Espírito Santo (UFES), Vitória, Espírito Santo, Brazil; 4 Empresa Brasileira de Pesquisa Agropecuária (EMBRAPA), Embrapa Recursos Genéticos e Biotecnologia, Brasília, Distrito Federal, Brazil; 5 Instituto de Biociências, Departamento de Genética, Universidade Estadual Paulista Júlio de Mesquita Filho (UNESP), Botucatu, São Paulo, Brazil; 6 Instituto de Biociências, Campus do Litoral Paulista, Universidade Estadual Paulista Júlio de Mesquita Filho (UNESP), São Vicente, São Paulo, Brazil; 7 Marine Mammal Institute, Hatfield Marine Science Center, Oregon State University, Newport, Oregon, United States of America; 8 Departamento de Ciências Agrárias e Biológicas, Universidade Federal do Espírito Santo (UFES), São Mateus, Espírito Santo, Brazil; Universidad de los Andes, COLOMBIA

## Abstract

Spinner dolphins (*Stenella longirostris*, Gray 1828) are widely distributed in tropical waters around the world. Although they occur in large, pelagic groups in the Eastern Tropical Pacific, elsewhere in the Pacific they are found in small and genetically isolated populations associated with islands. This species is considered to be “Least Concern” (LC) by the World Conservation Union (IUCN). To assess genetic diversity and population structure of an island-associated population in the South Atlantic Ocean we surveyed 162 spinner dolphins throughout the Fernando de Noronha Archipelago of the northeast coast of Brazil using ten microsatellite loci and sequencing a 413-bp section of the mitochondrial DNA (mtDNA) control region. Eleven mtDNA haplotypes were identified and haplotype diversity (*h*) and nucleotide diversity (*π*) were 0.3747 and 0.0060, respectively. Median-Joining Network revealed the presence of two very divergent haplotypes and F-statistics indicated some heterogeneity between two sampling years. All microsatellite loci were polymorphic (*H*_*o*_: 0.767; *H*_*e*_: 0,764) but, revealed no detectable substructure. We also compared the mtDNA haplotypes from Noronha to 159 haplotypes representing 893 individuals from 14 locations worldwide. We found that the two common haplotypes from the Fernando de Noronha Archipelago were absent in all other populations. These comparisons showed that Noronha spinner dolphins are likely more differentiated than other island populations, suggesting that they form societies with strong site fidelity mediated by females.

## Introduction

The spinner dolphin (*Stenella longirostris*, Gray 1828) is widely distributed in tropical waters around the world [1–4]. This wide distribution is reflected by considerable intraspecific variation in several morphological characters and ecological parameters. Four subspecies are recognized: Gray’s spinner subspecies *(Stenella longirostris longirostris)*, generally associated to tropical islands of the Indian, Atlantic and Pacific Oceans; the dwarf subspecies (*Stenella longirostris roseiventris)*, considerably small (140 cm), is found in waters of the Gulf of Thailand, Indonesia and southern China; the Central American subspecies (*Stenella longirostris centroamericana*), whose distribution covers coastal waters of the Pacific Ocean in Central America; the Eastern subspecies (*Stenella longirostris orientalis*), a morphologically distinct form in the Eastern Pacific Ocean, is found in tropical and coastal waters of Mexico [5,6].

Gray’s spinner dolphins (here after referred to as spinner dolphins) are found in oceanic waters of the Atlantic, Pacific and Indian oceans, and around volcanic islands and atolls, such as those of Hawaii, French Polynesia and Fernando de Noronha [7–9]. These dolphins can travel great distances (from 300 to 1000 miles) and are able to disperse between islands and atolls [10–13]. Large dispersal could have contributed to the high levels of genetic divergence exhibited by this species (around the world) [2–4,14].

Gray’s subspecies are the only spinner dolphin ecotype known to inhabit the South Atlantic Ocean [5,6]. Along the Brazilian coast the distribution of the spinner dolphin covers almost the entire length of the country [15–17]. The distribution reach 30°S, in waters up to 1000 m deep with low productivity and along the continental shelf break [15–17]. The temperature of these waters range from 24°C to 28°C, seeming to be displaced when the warm Brazil Current meets the cold Malvinas Current [15–17]. The Fernando de Noronha Archipelago, north-eastern Brazil, is considered a natural refuge of the species, where large groups of 600 individuals on average are observed daily [18].

Gray’s ecotype from the Fernando de Noronha Archipelago (hereafter referred to as Noronha spinner dolphins) gather in these islands to use their calm waters during the day to rest, play, care for their young and, take refuge from sharks; at night, they move out into deep sea waters to feed [17, 19]. While in the Pacific Ocean there are several rest areas along the coast of the Islands and even within atolls [20], in the Atlantic Ocean the Fernando the Noronha Archipelago encompasses the only area in the South West Atlantic Ocean available with resources and resting areas to support the large number of spinner dolphins [18].

The social structure of the spinner dolphin is considered to be transient, with groups merging and splitting up continuously, forming the so-called "fission-fusion" groups [21] in which the roles alternate between individuals and coalitions or groups [22]. Noronha spinner dolphins display two particularities recorded through photographs and videotapes. First, they probably use two distinct feeding zones at night, one closer to the archipelago (east side route) and other more distant (west side route). Second, they form distinct groups for copulation, a smaller group consisting of two to six males and one female, like the Hawaiian spinner dolphins, and a larger group formed by 50 to 100 individuals (many males and females), who display different behaviors like loud whistles, clicks, burst pulsed signals, and sometimes aggressive behavior, as follows: opening their beaks, biting and bumping each other, and leaving distinct scratches on the skin [23].

Despite the wide occurrence of spinner dolphins around the world, there are relatively few studies of genetic structure of island-associated populations worldwide and none in the South Atlantic Ocean [2–4, 24–26]. Molecular markers such as mitochondrial DNA sequences and microsatellite loci have been widely used in studies of genetic diversity, and have aided in the development of strategies for the conservation of animal species by determining the conservation status and the viability of natural populations [3, 27–30].

Moderate to high levels of variability generally confer a higher probability of population survival in a medium and long term [31]. Endangered species populations are small and often isolated, which can lead to a consequent loss of variability through reduced gene flow [31]. Based on intrapopulation genetic variability it is possible to estimate the effective population size, both throughout the evolutionary history of that population and in the more recent past (later generations, also called “contemporary”); to analyze demographic fluctuations over time; to test scenarios of population expansion or contraction [31]. Several factors may influence the structuring of dolphin individuals in different populations, such as the distribution of food resources (small fish) [32,33], social behavior [34], use habitats [35], and habitat discontinuities caused by environmental characteristics [36, 37].

It is very important to have a good knowledge of the basic biology of the species, including the degree of genetic variability, as well as the spatial and temporal aspects of intraspecific population structuring in order to develop appropriate strategies for the conservation and management of the populations [38]. *Stenella longirostris* is considered to be “Least Concern” (LC) by the World Conservation Union (IUCN, Red List of Threatened Species 2019), however, due to the scarcity of information regarding the genetics and geographical distribution patterns of *S*. *longirostris* in the Atlantic Ocean studies that focus on these issues are necessary.Here, we examine sequences of mitochondrial DNA (mtDNA) and 10 microsatellite loci to evaluate the genetic diversity of this species in the Fernando de Noronha Archipelago, and test the hypothesis of the existence of genetic structuring in the South Atlantic Ocean and haplotypes sharing among individuals from different oceans.

## Materials and methods

### Study area and sample collection

**Fig 1:**
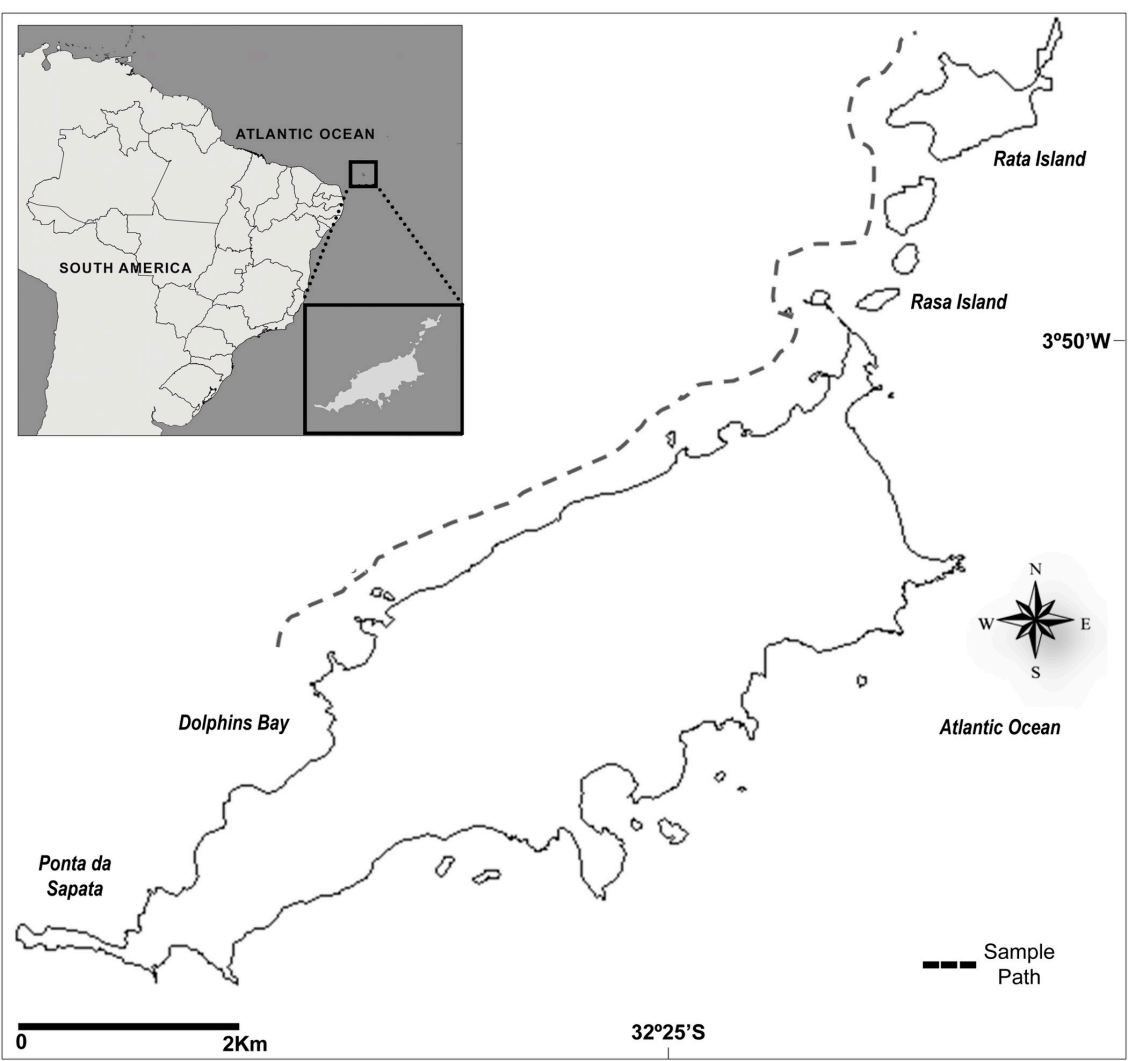
Fernando de Noronha Archipelago, coast of Brazil. Dashed lines indicate the path taken by spinner dolphins during the collection of the skin samples utilized in the study.

The Fernando de Noronha Archipelago is composed of 17 islands, located in the South Atlantic Ocean, 545 km apart of the Brazilian coast with a total area of 26 km^**2**^ [39]. These islands already occur in an area that involves the Area of Environmental Protection and the Marine National Park of Fernando de Noronha. The main island has 17 km^**2**^ distributed along the northeast-southwest direction (Fig 1). The archipelago is located at the fork of the South Equatorial current, which runs westbound with waters characterized by high salinity, low concentrations of sediments, organic matter, nutrients and plankton [39], high transparency, with depth of 87 m light extinction and averaged temperature of 27°C [40].

A total of 162 skin samples were collected from alive Noronha spinner dolphins by the skin swabbing method [41,42] in four different years: 2004 (N = 1); 2006 (N = 35); 2009 (N = 67); 2012 (N = 59) (Fig 1). The skin swabbing method is a non-invasive method that do not stress the animals much like other techniques of acquisition of biological material used for cetaceans that pierce the body of the alive animal [41,42]. This method uses sponges composed of synthetic fiber and abrasive material with a size of 8 X 8 cm fixed to a wooden mast (sampler) of 130 cm in length. One of the researchers with the sampler in hand lied face down on the bow of a small boat, and, with the approach of the dolphin, rubbed the sponge on the back or flank of the animal as it approaches of the bow of the vessel collecting skins that easily detach from the animal's body [41,42]. Immediately after contact, still in the vessel, the skin sample were removed from the sampler and stored in a microtube containing 70% alcohol [41,42]. The samples were sent and stored at -20°C in the Laboratório de Genética e Conservação Animal, Universidade Federal do Espírito Santo. *Stenella longirostris* is not an endangered or protected species, it is considered to be “Least Concern” by the World Conservation Union (IUCN, Red List of Threatened Species 2017). Licenses to transport and manipulate biological material collected at the Fernando de Noronha Archipelago were provided by the “Sistema de Autorização e Informação em Biodiversidade (SISBIO)/ Instituto Chico Mendes de Biodiversidade (ICMBio)”.

### Dataset of different oceans

**Fig 2:**
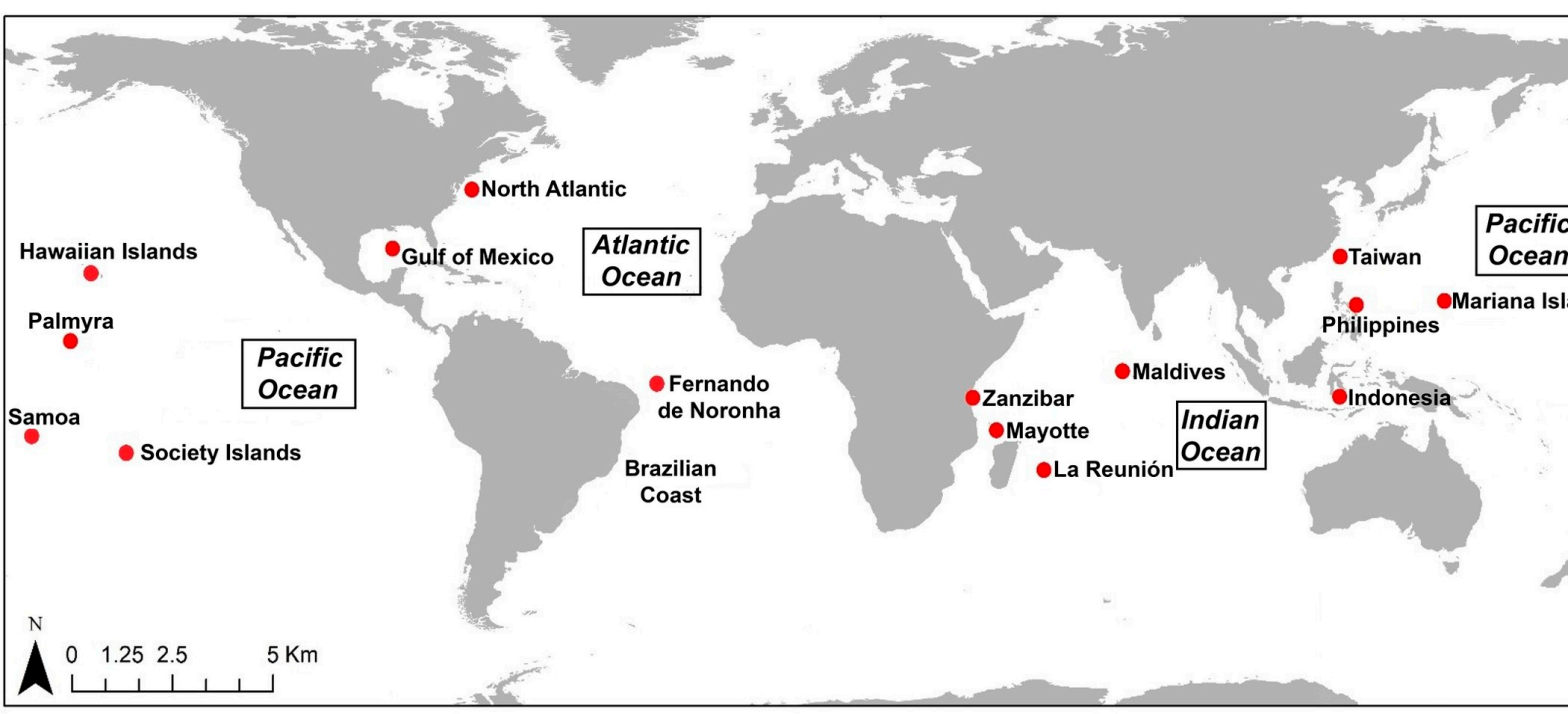
Map showing sampling locations for spinner dolphins analyzed in this study.

To compare the mtDNA haplotypes of the Noronha spinner dolphins worldwide, excluding the samples collected in Fernando de Noronha Archipelago, we reconstructed haplotypes from a total 159 sequences available in GenBank from five published papers (www.ncbi.nlm.nhi.gov/Genbank) [2–4, 26, 43]. These haplotypes represent 893 specimens of Gray’s spinner subspecies of 19 different locations of the world (Hawaiian Islands, Society Islands, 3-Islands, Guam, Saipan, Rota, American Samoa, Samoa, Zanzibar, Mayotte, La Reunión, Palmyra, Philippines, Maldives, Indonesia, Gulf of Mexico, Taiwan and North Atlantic) (Fig 2) (S1 Table).

To reduce the complexity of the comparisons some locations were pooled, reducing from 19 island samples to 14 locations in total: Fernando de Noronha, Hawaiian Islands, Society Islands, Mariana Islands (3-Islands + Guam + Saipan + Rota), Samoa (American Samoa + Independent Samoa), Zanzibar, Mayotte, La Reunión, Palmyra, Philippines, Maldives, Indonesia, Taiwan, and North Atlantic (Gulf of Mexico + North Atlantic).

### DNA extraction and sex determination

Genomic DNA was extracted from skin samples using Chelex resin (SIGMA) according to manufacturer's instructions. Since it is not possible to determine the sex of the animal during the skin swabbing method due to the impossibility of visualization of the genital organ of the living animal in the water during the dolphin displacement, the sex of each Noronha spinner dolphin was molecularly determined [44] by the amplification of ZFX and SRY genes using primers ZFX0582 and ZFX0923 [45]; PMSRYF [46] and TtSRYR [47]. The PCR reactions were carried out with the following modifications: 1 x Reaction Buffer, 150 μM of each dNTP, 1.5 mM MgCl_2_, 1.5 u of *Taq* polymerase (INVITROGEN) and 0.3 μM each primer, with the exception of the reverse for the SRY (0.06 μM) [47]. The final volume of the reaction was 25 μL [47]. The amplification was carried out under the following conditions: 92°C for 30 sec, followed by 35 cycles of 94°C for 30 sec, 51°C for 45 sec and 72°C for 45 seconds [47]. The fragments were separated on an 2.5% agarose gel, stained with GelRed (Biotium) [47].

### Microsatellite loci and analyses

Ten microsatellite loci were evaluated, of each seven specific of *S*. *longirostris*, (Table 1). Amplification reactions were carried out with the multiplex PCR Kit from Qiagen, using 1.5 μL of DNA and 0.1 μM of each primer in a final volume of 5 μL. Cycle conditions were: 95°C for 15 min, followed by 35 cycles of 94°C for 4 min, annealing temperature for 1 min and 30 s, and 72°C for 1 min and 30s, followed by a final 72°C extension for 15 min. Anneling temperatures for each of the primer pairs are listed in Table 1.

**Table 1:** Microsatellite loci used to access genetic diversity of spinner dolphin (*Stenella longirostris*). Loci names (Loci); primers sequences (Sequence 5’-3’), forward (F) and reverse (R) sequences; range of amplified fragments size (base pairs, bp); fluorescent primer labeling (Dye); annealing temperature (T°C); and Multiplex PCR reaction. Reference of original loci development: ^1^Galver, 2002; ^2^Farro, 2006; ^3^Amos et al., [48]; ^4^Valescchi & Amos [49].

Loci	Sequence 5’-3’	pb	Dye	T (°C)	Multiplex
^1^SD8	F: TGGCCGTTATAAATAGAGC	185–218	Fam	52°C	A
	R: GACAACAGTTTGGCAGTG				
^1^SL1-25	F: TTGATTTTCTGACTTCTTGGG	109–125	Fam	54°C	B
	R: CTCCGATATTGCCTTTACC				
^1^SL8-49	F: CATCTGTTCTTTGAATAGAGG	138–168	Hex	52°C	C
	R: ACCCATTCTGGTTCACC				
^1^SL9-69	F: TTCCAAACATACCCCTGCC	109–125	Hex	54°C	B
	R: ACTAGATGCCACTTGCACC				
^1^SL10-26	F: GCTATGTTATATCTATCTTCC	128–172	Hex	52°C	A
	R: TTAGGGCATTAATTTGAGTGC				
^2^SLO15	F: CGTCAAACTCCATCAAGACATC	218–252	Fam	52°C	D
	R: ATCTCCACCACAAGACACCAC				
^2^SLO1	F: CAAACCAAAAGCAAACACACAC	140–166	Fam	54°C	B
	R: CATCTCTATCAGCCATGTCCAA				
^3^415–416	F: GTTCCTTTCCTTACA	222–234	Fam	50°C	E
	R: ATCAATGTTTGTCAA				
^4^EV1	F: CCCTGCTCCCCATTCTC	115–197	Fam	52°C	F
	R: ATAAACTCTAATACACTTCCTCCAAC				
^4^EV94	F: ATCCTATTGGTCCTTTTCTGC	198–261	Hex	52°C	D
	R: AATAGATAGTGATGATGATTCACACC				

Capillary electrophoresis of PCR products was performed on ABI 3730 sequencer (Applied Biosystems). Fragment sizes were identified using GeneMapper software 5.0 (Applied Biosystems). The average non-exclusion probability for the identity of individuals was assessed using Cervus 3.0.3 [50] and potential duplicate samples were searched by comparing their genotypes.

Linkage disequilibrium and Hardy-Weinberg (HWE) equilibrium were evaluated using GENEPOP on the Web [51,52]. Microsatellite loci were also tested for the presence of null alleles and/or genotyping errors, using the Microchecker 2.2.0.3 [53], with Bonferroni correction.

Only the polymorphic loci were used in the analyses of genetic diversity. Total number of alleles (*Na*), mean number of alleles (*Nam*), number of private alleles (*Nap*), observed (*H*_*o*_) and expected (*H*_*e*_) heterozygosity and inbreeding coefficient (*F*_*IS*_) were calculated using Fstat v.2.9.3.2 [54] and Arlequin 3.5.2.2 [55]. Contemporary effective population size (Ne) was estimated using a method based on linkage disequilibrium [56,57] as implemented in NeEstimator 2.0 [58]. A *P*_*crit*_ value of 0.02 and 95% confidence intervals was chosen to reduce the potential bias for low frequency alleles [39]. A test for past or recent events of population bottleneck based on allele frequencies were performed using the software Bottleneck 1.2.02 [59]. The one-tailed Wilcoxon sign-rank test for heterozygote excess was used under the stepwise mutational model (SMM) [60] and the two-phase model (TPM; 30 variations, mutational model 70 gradual, 10000 interactions) [61]. A probability level of 0.05 was considered significant [62].

Temporal population structuring among the Noronha spinner dolphins sampled during the years 2006, 2009 and 2012 was investigated using Arlequin 3.5.2.2 [55]. The year 2004 was not analyzed due to just one individual sampled. Pairwise *F*_*ST*,_ based on number of differences, was estimated with 10000 random generations.

Population structure was also investigated in a Bayesian clustering analysis to estimate the most probable number of populations using Structure 2.3.2 [63], using an admixture model without prior population information and a correlated allele frequencies model. The burn-in length was set at 10,000 steps, followed by 500,000 repetitions of Markov Chain Simulation and Monte Carlo (MCMC). Ten independent executions were performed for each value of K varying between one and ten to test the consistency of estimates of P (XK). The number of clusters that best fit in the data was assessed and visualized using Structure Harvester [64] (available at http://taylor0.biology.ucla.edu/structureHarvester/), a web-based program for collating results generated by the program Structure 2.3.2 [64].

### mtDNA sequences and analyses

An approximately 550bp fragment of the mtDNA control region (D-loop) was amplified with the following primers: KRAdLp 1.5 t-pro [24] and dLp5 [65]. Polymerase Chain Reactions (PCR) were performed in 12,5 μl total volumes containing 1X Reaction Buffer, 200 μM of each dNTP, 2.0 mM MgCl_2_, 0.5 units of *Taq* DNA polymerase (INVITROGEN), and 0.2 μM of each primer [3]. Cycle conditions were as follows: 95°C for 1 min; followed by 40 cycles of 94°C for 30 s, 54°C for 30 s, and 72°C for 30 s; followed by a final 72°C extension for 15 min [3]. Amplification products were sequenced in both forward and reverse directions, with an ABI 310 automated sequencer (Applied Biosystems), and the sequences were aligned and edited manually using the algorithm Muscle of MEGA 6.06 [66].

Arlequin 3.5.2.2 [55] was used to calculate nucleotide (*π*) and haplotypic (*h*) diversities and mismatch distribution, to test selective neutrality in mtDNA control region and population structuring. Selective neutrality in mtDNA control region was tested by the Tajima test [67] and Fu [68] using a coalescent simulation algorithm (10 000 steps).

Temporal population structuring was investigated among Noronha spinner dolphins sampled during the years 2006, 2009 and 2012, since the year 2004 was removed from this analysis due just one individual sampled. Pairwise *F*_*ST*_ and *Φ*_*ST*_ were estimated with 10,000 random generations using Arlequin 3.5.2.2 [55].

Genetic differentiation was also assessed among Noronha spinner dolphins and other locations of the world using AMOVA, Pairwise *F*_*ST*_ and Ф_*ST*_ using Arlequin 3.5.2.2 [55], using haplotype frequencies only and pairwise difference, respectively. A Median-Joining analysis among haplotypes was estimated through Network 4.5 [51] (http://www.fluxus-enginering.com), and the MP option was used to reduce the complexity [69].

## Results

### Noronha spinner dolphins genetic diversity and population structure

#### Sex identification

From the analysis of 162 individuals, we identified the sex of 137 individuals (84 males and 53 females). The remainder of samples failed to amplify.

#### Microsatellite loci

Of the 162 skin samples, 92 provided genotypes for 7–10 loci. Three loci were not used in the analysis: SL8-49 was monomorphic; SL10-26 revealed the presence of null alleles; and SL01 was discarded because of evidence of linkage disequilibrium. The seven other microsatellite loci were moderately variable, e.g. k = 4–15 (Table 2). The probability of identity was 1, 6 X 10^−9^. None of the samples had matching genotypes.

**Table 2:** Genetic diversity of Noronha spinner dolphins (*Stenella longirostris*) for seven microsatellite loci. *N*: sample size for each locus; *Nak*: total number of alleles; *H*_*O*_: observed heterozygosity; *H*_*e*_: expected heterozygosity; *PIC*: polymorphic information content; *Ra*: allelic richness; *Dg*: genetic diversity; *F*_*IS*_: inbreeding coefficient; *F (Null)*: estimation of frequency of null alleles); Hardy-Weinberg Equilibrium P (HWE).

Loci	*N*	Na	*H*_*o*_	*H*_*e*_	*PIC*	*Ra*	*F*_*IS*_	F(Null)	P(HWE)
415–416	76	9	0.763	0.801	0.767	8.835	0.047	+0.019	0.203
EV1	66	12	0.758	0.825	0.795	12.000	0.082	+0.044	0.079
EV94	81	13	0.840	0.841	0.818	12.774	0.002	+0.000	0.293
SD8	91	15	0.846	0.829	0.805	13.878	-0.021	-0.013	0.029*
SLI-25	91	10	0.813	0.758	0.732	9.904	-0.073	-0.045	0.019*
SL9	90	7	0.756	0.701	0.646	6.663	-0.078	-0.044	0.011*
SLO15	83	4	0.590	0.594	0.529	3.959	0.006	+0.008	0.733
Mean	82,57	10	0.767	0.764	0.727	9.716			

(*) Statistically significant values for HWE P < 0.05.

Three loci showed significant departure from Hardy Weinberg equilibrium, with a deficiency in homozygotes (Table 2).

Using NeEstimator, contemporary effective population size (Ne) for Noronha spinner dolphins was estimated to be 491.3 (95% CI 153.1 - ∞; harmonic mean sample size: 70.8). The Wilcoxon one-tailed test revealed that the Noronha spinner dolphins went through a possible bottleneck event based on the TPM mutational model (P = 0.00391), but not under SMM model (P = 0.3475).

**Fig 3:**
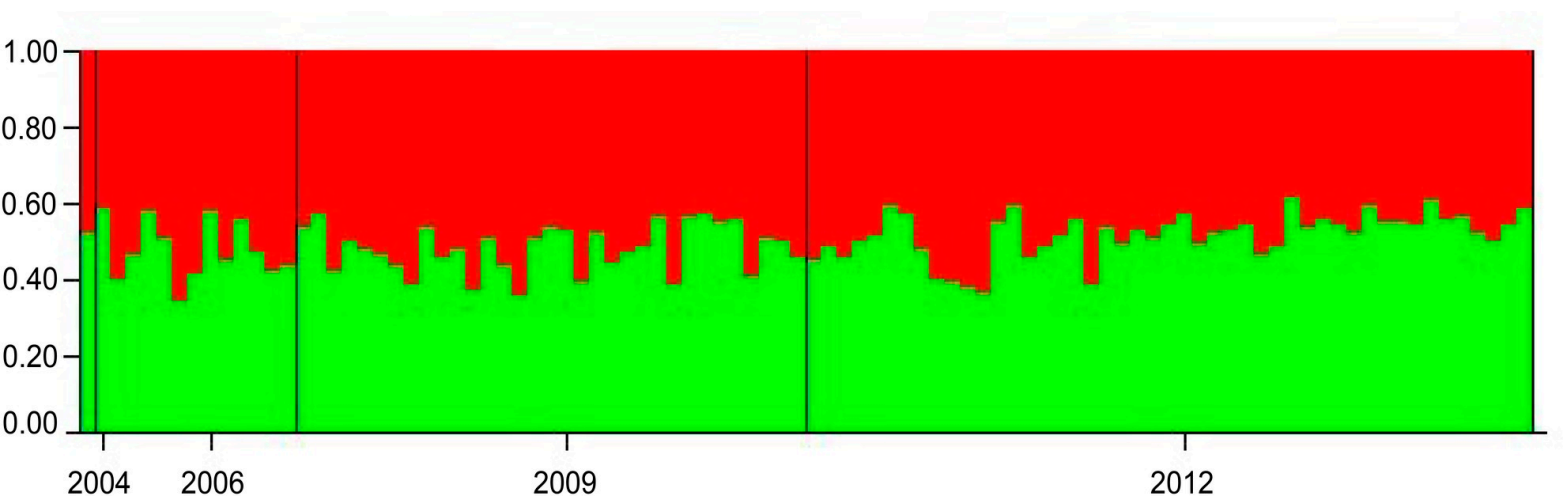
Bayesian clustering analysis inferred with the program structure 2.3.2 for seven microsatellite loci of Noronha spinner dolphins (*Stenella longirostris*). Assignment probabilities of individuals to putative population clusters at k = 2 according to sampling year (2004, 2006, 2009, 2012).

The temporal genetic structuring test applied between individuals sampled in different years showed no statically significant *F*_*ST*_ values. The Bayesian clustering analysis revealed no detectable substructure by sampling period or overall. The greatest likelihood was K = 1, and, K = 2 showed all individuals to have similarly mixed ancestries (Figs 3 and S1).

Analysis excluding the three loci that showed significant departure from Hardy Weinberg equilibrium (SD8, SLI25, SL9) displayed the same pattern of results as the analysis containing them: no statically significant *F*_*ST*_ values; Ne = 271.5 (95% CI 63.6 - ∞; harmonic mean sample size: 60.5); no bottleneck event, TPM mutational model (P = 0.96875), SMM model (P = 0.06250); no detectable substructure by sampling period or overall (S2 Fig).

#### mtDNA

Sequences of a 413 bp fragment of the mtDNA control region were obtained for all 162 samples of Noronha Spinner dolphins and revealed 27 variable sites (20 transitions and eight transversions) representing 11 haplotypes (defined as NOR), uploaded to GenBank with accession numbers: MK184992 –MK185002. Haplotype diversity (*h*) and nucleotide diversity (*π*) were 0.374 (+/- 0.044) and 0.006 (+/- 0.003), respectively.

**Fig 4:**
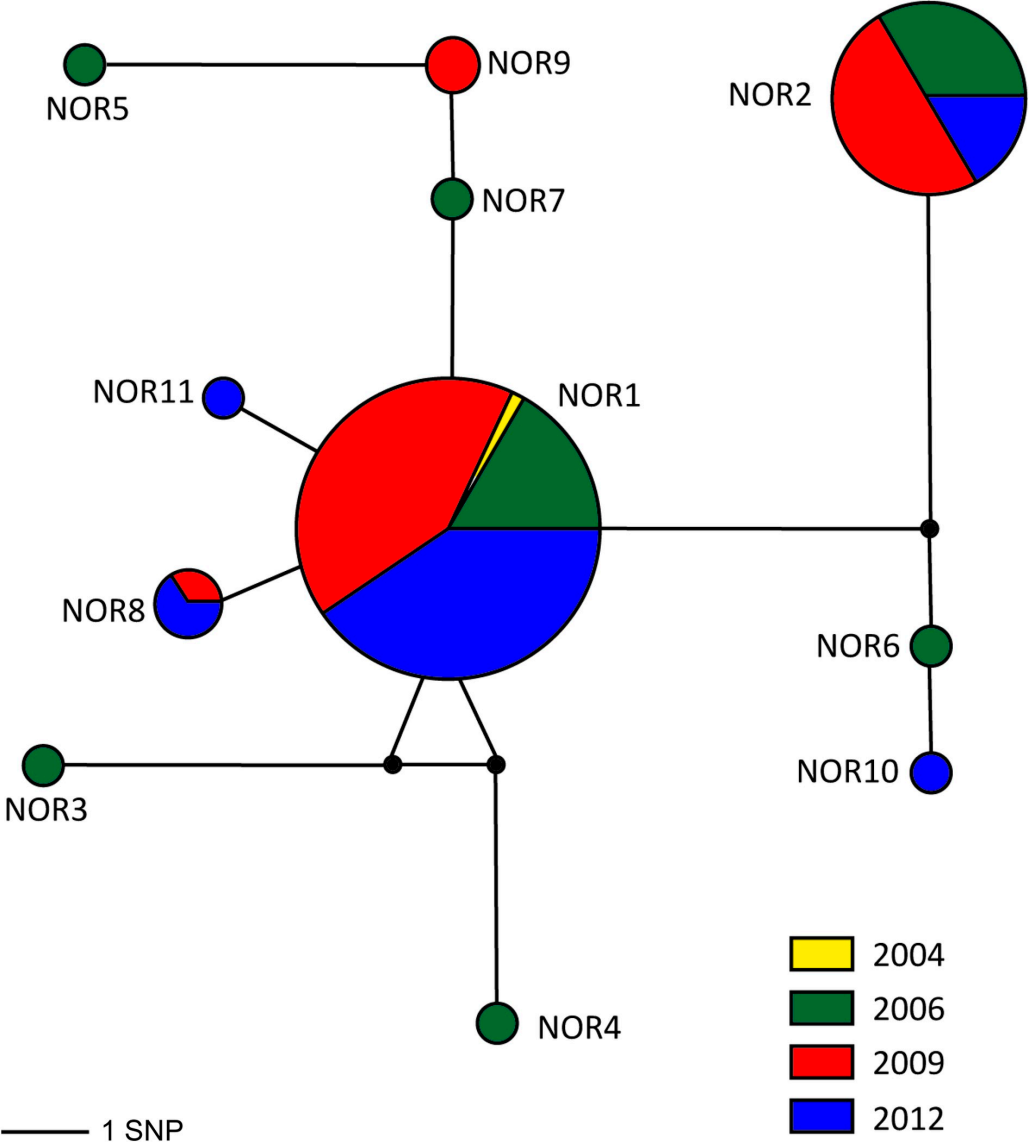
Temporal Median-Joining network including the individuals from 2004, 2006, 2009 and 2012 from the Noronha spinner dolphins (*Stenella longirostris*) (FN). Each circle corresponds to a haplotype, and its size is proportional to its frequency in the population. Black circles indicate potential intermediate haplotypes not sampled. Different colors represent the sampling years. Lengths of lines connection haplotypes are proportional to the number nucleotide differences between haplotypes.

Median-Joining network showed two very divergent haplotypes: NOR1 (N = 126) and NOR2 (N = 24); NOR1 was shared among individuals from 2004, 2006, 2009 and 2012; NOR2 was shared among the individuals of 2006, 2009 and 2012; NOR2 is distant from the majority of haplotypes by eight mutational steps (Fig 4).

The mismatch distribution chart of the Noronha spinner dolphins mtDNA presented a bimodal curve (S3 Fig). The neutrality tests were not significant: Fu’s *Fs* (Fs = 0.177, *P* < 0.589) and Tajima’s *D (*D = -1.369, *P* < 0.055). The temporal genetic structuring test applied for individuals sampled in different years showed significant *F*_*ST*_ values for mtDNA control region between 2006 and 2012 (*F*_*ST*_: 0.087; *P* ≤ 0.006).

### Worldwide *S*. *longirostris* genetic diversity, population structure and phylogeography

To investigate the phylogeographic relationship of Noronha spinner dolphins to other populations worldwide, we analyzed a total of 1,055 samples representing 14 regional populations (S1 Table), including 162 samples from Noronha spinner dolphins and 893 samples from the other 14 locations. The total length of sequences varied from 374 to 555 bp, allowing a consensus length of 374 bp for all analyses, resolving 158 haplotypes. At the consensus length of 374 bp, the Noronha spinner dolphins showed nine haplotypes (i.e., 2 haplotypes collapsed).

Haplotypic diversity of the 14 populations ranged from (*h =* 0.345) for Noronha spinner dolphins (despite the large number of specimens) to (*h* = 0.953) for Mayotte, whereas nucleotide genetic diversity ranged from (*π =* 0.005) for Hawaiian Islands to (*π* = 0.022) for Samoa (Table 3). Noronha spinner dolphins showed the second lowest nucleotide diversity (*π* = 0.006).

**Table 3:** Samples size and genetic diversity values for 374 pb of mtDNA control region by location analyzed for spinner dolphins (*Stenella longirostris*). *Ni*: number of individuals; *Nh*: number of haplotypes; *h*: haplotype diversity; *π*: nucleotide diversity from the Noronha spinner dolphins. GB: specimens from GenBank.

*mtDNA* (374bp)					
Locations	*Ni*	*Nh*	*h*	*π*	Source
1. Fernando de Noronha	162	9	0.345	0.006	This study
2. Hawaiian Islands	501	19	0.507	0.005	GB (Andrews et al. 2010)
3. Society Islands	154	29	0.922	0.018	GB (Oremus et al. 2007)
4. Mariana Islands	103	25	0.928	0.017	GB (Andrews et al. 2013; Martien et al. 2014)
5. Samoa	32	14	0.951	0.022	GB (Andrews et al. 2010; Andrews et al. 2013)
6. Zanzibar	29	12	0.864	0.018	GB (Viricel et al. 2016)
7. Mayotte	19	13	0.953	0.018	GB (Viricel et al. 2016)
8. La Reunión	16	9	0.900	0.016	GB (Viricel et al. 2016)
9. Palmyra	10	7	0.911	0.010	GB (Andrews et al. 2013)
10. Philippines	7	6	0.952	0.021	GB (Andrews et al. 2013)
11. North Atlantic	7	4	0.714	0.015	GB (Andrews et al. 2013)
12. Maldives	6	4	0.866	0.020	GB (Andrews et al. 2013)
13. Indonesia	5	4	0.900	0.011	GB (Andrews et al. 2013)
14. Taiwan	4	3	0.833	0.012	GB (Andrews et al. 2013)
Total	1055	158			

*F*-statistics of mtDNA control region among spinner dolphins of different locations revealed significant differences among almost all locations and among all ocean basins (Table 4).

**Table 4:** Pairwise *F*_*ST*_ (lower diagonal) and Φ_*ST*_ (upper diagonal) for spinner dolphins for mtDNA control region among locations of the world.

	1	2	3	4	5	6	7	8	9	10	11	12	13	14
1. F. Noronha	-	0.486***	0.321***	0.263***	0.379***	0.540***	0.482***	0.571***	0.354**	0.528***	0.511**	0.562***	0.572***	0.482**
2. Hawaiian I.	0.550***	-	0.432***	0.412***	0.486***	0.647***	0.585***	0.676***	0.488***	0.658***	0.644***	0.556***	0.642***	0.566***
3. Society I.	0.368***	0.316***	-	0.035***	0.011	0.152***	0.097***	0.168***	0.047	0.100*	0.065	0.098*	0.101*	0.066
4. Mariana I.	0.389***	0.316***	0.056***	-	0.010	0.172***	0.107***	0.186***	0.011	0.127*	0.086*	0.126*	0.142**	0.053
5. Samoa	0.447***	0.335***	0.039***	0.039***	-	0.134***	0.079**	0.148***	0.010	0.055	0.054	0.069	0.094*	0.012
6. Zanzibar	0.487***	0.377***	0.103***	0.100***	0.091***	-	-0.005	0.003	0.230***	0.063	0.053	0.259**	0.151*	0.250**
7. Mayotte	0.472***	0.349***	0.063***	0.059**	0.047***	0.017	-	0.032	0.173**	0.030	0.001	0.203**	0.140*	0.195*
8. La Reunión	0.466***	0.372***	0.087***	0.084***	0.072***	0.113***	0.072***	-	0.282**	0.061	0.089	0.349***	0.186*	0.318**
9. Palmyra	0.514***	0.373***	0.071**	0.063**	0.048*	0.111***	0.066**	0.094**	-	0.201*	0.177*	0.182**	0.239***	0.130
10. Philippines	0.516**	0.369***	0.063*	0.061*	0.048*	0.098*	0.047*	0.076*	0.055	-	0.064	0.267*	0.128	0.151
11. N. Atlantic	0.545***	0.401***	0.098**	0.095**	0.082**	0.103*	0.067*	0.114**	0.109*	0.089*	-	0.149	0.145*	0.207*
12. Maldives	0.545***	0.396**	0.083*	0.082*	0.068*	0.122*	0.068*	0.100*	0.093	0.071	0.117	-	0.250**	0.119
13. Indonesia	0.580***	0.441***	0.154***	0.152***	0.143***	0.194***	0.147***	0.179**	0.180**	0.166*	0.212*	0.201*	-	0.228*
14. Taiwan	0.570**	0.420**	0.107**	0.104*	0.090**	0.146**	0.091*	0.124*	0.119	0.098	0.148	0.131	0.236*	-

Statistically significant values are highlighted in gray and have asterisks:

*P < 0.05,

**P < 0.01,

***P < 0.001.

Significant differentiation was observed between Noronha spinner dolphins and all other locations of the world; the highest (F_*ST*_ = 0.580) value was observed with Indonesia, and the lowest (F_*ST*_ = 0.368) with Society Islands (Table 4). Overall, the highest level of differentiation was observed between Hawaiian Islands and La Reunión (F_*ST*_ = 0.676) and the lowest between Mariana Islands and Society Islands (F_*ST*_ = 0.035). The AMOVA analysis revealed significant values among locations (F_*ST*_ = 0.341, *P*<0.001).

**Fig 5:**
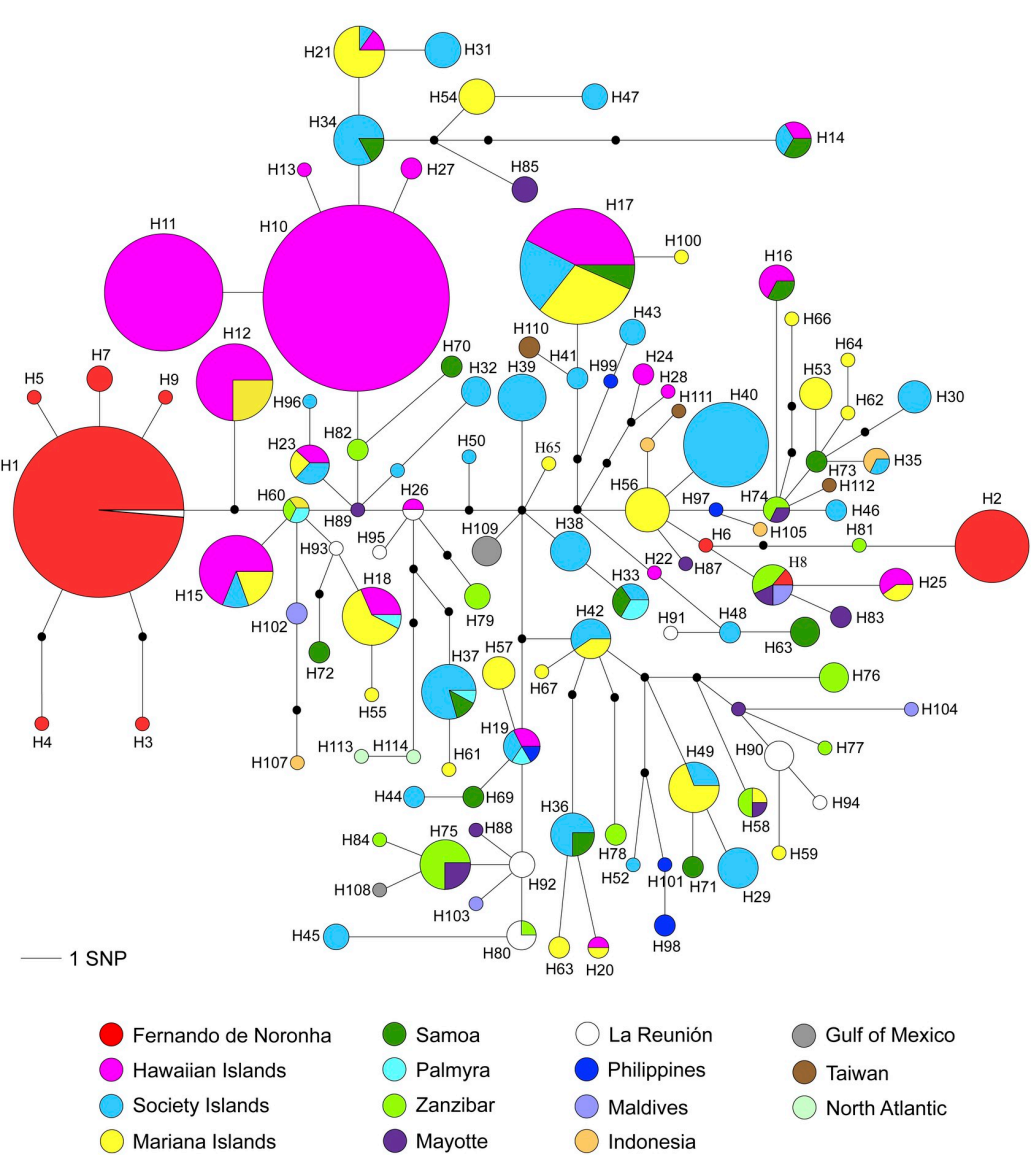
Median-Joining of mtDNA control region haplotypes for spinner dolphins (*Stenella longirostris*) worldwide. Each circle corresponds to a haplotype, and its size is proportional to its frequency in the population. Black circles indicate potential intermediate haplotypes not sampled. Different colors represent the sampling years. Lengths of lines connection haplotypes are proportional to the number nucleotide differences between haplotypes.

The haplotype network for spinner dolphin mtDNA control region of different locations around the world emphasizes the divergence and distinctiveness of the H1 and H2 haplotypes in Noronha showing that these dolphins are more differentiated than other island populations in the Pacific and Indian Oceans (Fig 5). H1 encompasses the majority of Noronha spinner dolphins with other haplotypes (H3, H4, H5, H7, H9), represented by one or two specimens around it. H1 and H2 are closest to other haplotypes from geographically very distant locations than each other. The H2, H6 and H8 are very distant from all other haplotypes from Fernando de Noronha, at least nine mutation steps of separation (Fig 5). The haplotype network also showed no phylogeographic signal among the locations of the world, and that Noronha spinner dolphins share two haplotypes with other island populations of the world: the H1 shared with one sample from La Reunión and H8 represented by one sample from Noronha, three samples from Zanzibar, one sample from Mayotte, and two samples from Maldives (Fig 5).

## Discussion

Our analyses provide the first valuable insights into genetic diversity and differentiation of Gray’s spinner dolphins of the Southwest Atlantic Ocean. Our study also presents one of the most complete analyses of mtDNA structure and diversity of spinner dolphins in terms of sample size (893 individuals) and phylogeographic coverage (15 populations) crossing 3 ocean basins. Understanding the degree of genetic variability, as well as the spatial and temporal aspects of its intraspecific population structure, is necessary for the implementation of adequate conservation and management strategies. Particularly, the lack of regional genetic study for spinner dolphin, does not allow asses population structure and consequently no effectively regional management plan has been implemented. The ecotourism industry of spinner dolphin observation in Fernando de Noronha Archipelago is a growing concern that can be impacting the dynamic of this population.

Here we used mitochondrial DNA (D-loop) and nuclear microsatellite loci to assess the patterns of genetic diversity and population structure of the spinner dolphins of Fernando de Noronha Archipelago, Southwest Atlantic Ocean. Mitochondrial DNA revealed low genetic diversity in relation to other population worldwide (Table 3), high differentiation, in terms of pair-wise *F*_*ST*_, and high isolation from other populations around the world. These findings emphasize spinner dolphin vulnerability to genetic processes and highlight the importance of the development of conservation programs for this population.

Noronha spinner dolphins revealed the lowest level of mtDNA diversity of any spinner dolphin population around the world [3, 26, 2, 43, 4] and even among threatened odontocetes [70–73]. To our knowledge, the only report of a population of dolphins with lower mtDNA diversity is the Hector's dolphin (*Cephalorhynchus hectori*) of the north island of New Zealand [74].

However, low variability in the mitochondrial DNA control region does not necessarily reflects low levels of heterozygosity in the nuclear genome [75]. Indeed, microsatellites revealed moderate to high levels of heterogozity for Noronha spinner dolphins. Low values of mitochondrial genetic diversity were reported for spinner dolphins of the North-western Hawaiian Islands: Kure Atoll (N = 51, *h =* 0.395), Midway Atoll (N = 117, *h =* 0.405), Pearl & Hermes Reef (N = 47, *h =* 0.200) [3]. Small population sizes were estimated for islands by photo-identification studies for some Hawaiian Islands, Kure (≅120 individuals) and Midway (≅ 260 individuals), and were consistent with the scarcity of resting habitat and foraging habitat in this region [76]. This pattern seems not to be applicable to Noronha spinner dolphins (N = 162), which encompasses large numbers of individuals estimated daily by photo-identification studies (≅ 600 individuals) [18]. Instead, an alternative explanation would be low levels of immigration driven by social or demographic forces, as it has been seen in the Tahiti spinner dolphin [2]; or recent demographic bottleneck as demonstrated by significant microsatellite results.

The predominance of one haplotype of Noronha spinner dolphins can be explained by the founder effect. The bimodal curve of the mismatch distribution chart indicates that this population unit has undergone a recent demographic expansion [77,78] or through a scale expansion with high levels of migration between neighbors [79]. This analysis is usually multimodal for population in demographic balance, given that it reflects the highly stochastic gene trees. We can hypothesize that Noronha spinner dolphins originally had few mitochondrial lineages and that the effects of genetic drift would lead to loss of alleles (haplotypes). Additionally, the restricted gene flow (promoted mainly by females) between this population unit and the spinner dolphins of other localities of Brazilian coast, would not be sufficient to compensate the effects of genetic drift leading to loss of the rarer allele and the maintenance of the most frequent haplotype. However, further analyses are necessary to prove this hypothesis and the lack of information about spinner dolphins from other localities along the Brazilian coastline limits answers about the maintenance of gene flow between these stocks.

The presence of two distinctive and divergent haplotypes of Noronha spinner dolphin’s lineages also suggests colonization of this archipelago by at least two different matrilines. Comparisons among locations of the world indicate genetic isolation of Noronha spinner dolphins from all other spinner dolphin’s populations. This is likely due to the geographic isolation of this island from other islands of the Atlantic and Pacific Oceans. The Fernando de Noronha Archipelago encompasses the only islands in the Southwest Atlantic Ocean with resources and rest areas to support a large number of spinner dolphins [18]. Studies describe that spinner dolphins are found in every ocean [1–3] encompassing large groups of individuals [1,23] displaying the ability to travel for long distances and to dispersal between islands and atolls contributing to variations in the levels of disperse and genetic divergence around the world [10–13]. Our results indicate that these dolphins followed a mainland-island model of metapopulation dynamic, with pelagic populations representing the ‘mainland’ in the model [80].

Ocean water masses of Southwest Atlantic Ocean also exercise highly significant role on the distribution of spinner dolphins and can have important role in levels gene flow of spinner dolphins in the Brazilian coast. The Brazil Current and the Malvinas Current are considered as the limit of distribution of this species in southern Brazil encouraging the displacement of these dolphins to the south of the country in hot seasons, while in colder seasons these dolphins move to the north of the country [15,16].

The vicinities of Pernambuco, where the South Equatorial Current splits to the North Brazil Current and South Brazil Current, can be the limit of distribution of *S*. *longirostris longirostris* between Northeast and Southeast of Brazil. The South Equatorial Current can encourage the displacement of spinner dolphins of the Fernando de Noronha Archipelago towards the Brazilian coast [81]. This current can also influence the displacement of dolphins from the coast of Africa and Pacific Ocean to the Brazilian coast explaining the shared haplotypes among Noronha spinner dolphins and La Reunión, Maldives and Zanzibar. Climate can impact the ocean water masses displacement and cause habitat changes and alterations in the gene flow of small cetacean population units [82,83].

Some evidence of heterogeneity in the Noronha population was suggested by annual difference in mtDNA frequencies, although not in microsatellite. This could be explained by feeding behavior possibly inherited matrilineally once recent observations suggest two different groups of spinner dolphins that appear to use distinct feeding strategies at Fernando de Noronha Archipelago [23]. Typically, spinner dolphins use the calm waters of the archipelago during the day to rest, play, care for their young, take refuge from sharks, and during the night moving to sea to feed [23]. However, in Fernando de Noronha Arquipelago, observations of the same dolphins coming to the archipelago in several days and the long periods of absence of other dolphins, can indicate two distinct feeding strategies: “around the archipelago” (in a radius of up to 5 nautical miles), and “oceanic” (on the slopes of mountains or submerged underwater geological chain of oceanic region adjacent to Fernando de Noronha) [23].

We showed that Noronha spinner dolphins display low levels of genetic diversity and that the colonization of Archipelago was driven by at least two different matrilineal lines. We also suggest that these dolphins live in societies mediated by females, with strong geographic fidelity and greater genetic isolation in comparison to other island populations from different ocean basins.

## Supporting information

S1 Table Haploypes from GenBank used in this study. Table includes source, GenBank accession number, haplotypes codes of the original papers and of the present study, and, geographic localizations with number of individuals.
(DOCX)

S1 Fig Bayesian clustering analysis inferred with the program Structure 2.3.2 for seven microsatellite loci of Noronha spinner dolphins (Stenella longirostris): (a) ΔK from Evanno’s method is shown between successive K values, (b) mean log probability (Lk) is given for each K tested.
(TIF)

S2 Fig Bayesian clustering analysis inferred with the program Structure 2.3.2 for four microsatellite loci of Noronha spinner dolphins (Stenella longirsotris): (a) ΔK from Evanno’s method is shown between successive K values, (b) mean log probability (Lk) is given for each K tested, and the (c) assignment probabilities of individuals to putative population clusters at K = 2 according to sampling year (2004, 2006, 2009, 2012).
(TIF)

S3 Fig Mismatch distribution of Noronha spinner dolphins (*Stenella longirostris*). Observed and expected distributions under spatial expansion model are shown with bars and lines, respectively.
(TIF)
