# Supplementary material for: Low mtDNA diversity in a highly differentiated population of spinner dolphins (Stenella longirostris) from the Fernando de Noronha Archipelago, Brazil
Source: PLoS One. 2020 Apr 7;15(4):e0230660. doi: 10.1371/journal.pone.0230660 (PMC7138316; doi:10.1371/journal.pone.0230660)
Supplement: S1 Table — Table includes source, GenBank accession number, haplotypes codes of the original papers and of the present study, and, geographic localizations with number of individuals. (DOCX) [file pone.0230660.s001.docx]

## S1 Table. Haploypes from GenBank used in this study. Source, GenBank accession number, haplotypes codes of the original papers and of the present study, and, geographic localizations with number of individuals.

| **Source (Paper)** | **GenBank acession** | **Haplotype code (Original Paper)** | **Haplotype code**  **(This Study)** | **Geographic localization (nº of individuals)** |
| --- | --- | --- | --- | --- |
| Andrews *et al.* 2010 | GU253256 | HW01 | H 10 | Hawaii (344) |
| Andrews *et al.* 2010 | GU253257 | HW02 | H 11 | Hawaii (63) |
| Andrews *et al.* 2010 | GU253258 | HW03 | H 12 | Hawaii (20) |
| Andrews *et al.* 2010 | GU253259 | HW04 | H 13 | Hawaii (1) |
| Andrews *et al.* 2010 | GU253260 | HW05 | H 14 | Hawaii (2), Samoa (1) |
| Andrews *et al.* 2010 | GU253261 | HW06 | H 15 | Hawaii (18) |
| Andrews *et al.* 2010 | GU253262 | HW07 | H 16 | Hawaii (4), Samoa (2) |
| Andrews *et al.* 2010 | GU253263 | HW08 | H 17 | Hawaii (26), Samoa (2) |
| Andrews *et al.* 2010 | GU253264 | HW09 | H 18 | Hawaii (5) |
| Andrews *et al.* 2010 | GU253265 | HW10 | H 19 | Hawaii (2) |
| Andrews *et al.* 2010 | GU253266 | HW11 | H 20 | Hawaii (1) |
| Andrews *et al.* 2010 | GU253267 | HW12 | H 21 | Hawaii (2) |
| Andrews *et al.* 2010 | GU253268 | HW13 | H 22 | Hawaii (1) |
| Andrews *et al.* 2010 | GU253269 | HW14 | H 23 | Hawaii (3) |
| Andrews *et al.* 2010 | GU253270 | HW15 | H 24 | Hawaii (2) |
| Andrews *et al.* 2010 | GU253271 | HW16 | H 25 | Hawaii (3) |
| Andrews *et al.* 2010 | GU253272 | HW17 | H 26 | Hawaii (1) |
| Andrews *et al.* 2010 | GU253273 | HW18 | H 27 | Hawaii (2) |
| Andrews *et al.* 2010 | GU253274 | HW19 | H 28 | Hawaii (1) |
| Andrews *et al.* 2010 | GU253275 | HW20 | H 68 | Samoa (2) |
| Andrews *et al.* 2010 | GU253276 | HW21 | H 69 | Samoa (1) |
| Andrews *et al.* 2010 | GU253277 | HW22 | H 70 | Samoa (1) |
| Andrews *et al.* 2010 | GU253278 | HW23 | H 36 | Samoa (1) |
| Andrews *et al.* 2010 | GU253279 | HW24 | H 34 | Samoa (1) |
| Andrews *et al.* 2010 | GU253280 | HW25 | H 43 | Samoa (1) |
| Andrews *et al.* 2010 | GU253281 | HW26 | H 33 | Samoa (1) |
| Andrews *et al.* 2010 | GU253282 | HW27 | H 37 | Samoa (1) |
| Andrews *et al.* 2010 | GU253283 | HW28 | H 71 | Samoa (1) |
| Andrews *et al.* 2010 | GU253284 | HW29 | H 72 | Samoa (1) |
| Andrews *et al.* 2013 | KC160999 | StLo003 | H 15 | Guam (1), Saipam (2), Samoa (2) |
| Andrews *et al.* 2013 | KC161018 | StLo022 | H 60 | Palmyra (1) |
| Andrews *et al.* 2013 | KC161021 | StLo025 | H 74 | Zanzibar (2) |
| Andrews *et al.* 2013 | KC161037 | StLo041 | H 8 | Maldives (2), Zanzibar (2) |
| Andrews *et al.* 2013 | KC161038 | StLo042 | H 14 | American Samoa (1) |
| Andrews *et al.* 2013 | KC161041 | StLo045 | H 108 | Gulf of mexico (1) |
| **Source (Paper)** | **GenBank acession** | **Haplotype code (Original Paper)** | **Haplotype code**  **(This Study)** | **Geographic localization (nº of individuals)** |
| Andrews *et al.* 2013 | KC161042 | StLo046 | H 109 | Gulf of mexico (4) |
| Andrews *et al.* 2013 | KC161043 | StLo047 | H 62 | Guam (1) |
| Andrews *et al.* 2013 | KC161044 | StLo048 | H 63 | Guam (1) |
| Andrews *et al.* 2013 | KC161045 | StLo049 | H 57 | Guam (1) |
| Andrews *et al.* 2013 | KC161046 | StLo050 | H 54 | Guam (1), Saipan (1) |
| Andrews *et al.* 2013 | KC161047 | StLo051 | H 105 | Indonesia (1) |
| Andrews *et al.* 2013 | KC161048 | StLo052 | H 35 | Indonesia (2) |
| Andrews *et al.* 2013 | KC161049 | StLo053 | H 106 | Indonesia (1) |
| Andrews *et al.* 2013 | KC161050 | StLo054 | H 107 | Indonesia (1) |
| Andrews *et al.* 2013 | KC161051 | StLo055 | H 102 | Maldives (2) |
| Andrews *et al.* 2013 | KC161052 | StLo056 | H 103 | Maldives (1) |
| Andrews *et al.* 2013 | KC161053 | StLo057 | H 104 | Maldives (1) |
| Andrews *et al.* 2013 | KC161055 | StLo059 | H 113 | North atlantic (1) |
| Andrews *et al.* 2013 | KC161056 | StLo060 | H 114 | North atlantic (1) |
| Andrews *et al.* 2013 | KC161057 | StLo061 | H 18 | Saipam (1), Palmyra (1) |
| Andrews *et al.* 2013 | KC161058 | StLo062 | H 19 | Palmyra (1), Philippines (1) |
| Andrews *et al.* 2013 | KC161059 | StLo063 | H 97 | Philippines (1) |
| Andrews *et al.* 2013 | KC161060 | StLo064 | H 98 | Philippines (2) |
| Andrews *et al.* 2013 | KC161061 | StLo065 | H 99 | Philippines (1) |
| Andrews *et al.* 2013 | KC161062 | StLo066 | H 100 | Philippines (1) |
| Andrews *et al.* 2013 | KC161063 | StLo067 | H 101 | Philippines (1) |
| Andrews *et al.* 2013 | KC161064 | StLo068 | H 23 | Palmyra (3) |
| Andrews *et al.* 2013 | KC161065 | StLo069 | H 96 | Palmyra (1) |
| Andrews *et al.* 2013 | KC161066 | StLo070 | H 37 | Palmyra (1), American Samoa (1) |
| Andrews *et al.* 2013 | KC161067 | StLo071 | H 33 | American Samoa (1), Palmyra (2) |
| Andrews *et al.* 2013 | KC161068 | StLo072 | H 68 | American Samoa (2) |
| Andrews *et al.* 2013 | KC161069 | StLo073 | H 69 | American Samoa (1) |
| Andrews *et al.* 2013 | KC161070 | StLo074 | H 70 | American Samoa (1) |
| Andrews *et al.* 2013 | KC161071 | StLo075 | H 73 | American Samoa (2) |
| Andrews *et al.* 2013 | KC161072 | StLo076 | H 36 | American Samoa (1) |
| Andrews *et al.* 2013 | KC161073 | StLo077 | H 34 | American Samoa (1) |
| Andrews *et al.* 2013 | KC161074 | StLo078 | H 43 | American Samoa (1) |
| Andrews *et al.* 2013 | KC161075 | StLo079 | H 71 | American Samoa (1) |
| Andrews *et al.* 2013 | KC161076 | StLo080 | H 72 | American Samoa (1) |
| Andrews *et al.* 2013 | KC161077 | StLo081 | H 25 | Saipan (1) |
| Andrews *et al.* 2013 | KC161096 | StLo100 | H 110 | Taiwan (2) |
| Andrews *et al.* 2013 | KC161097 | StLo101 | H 111 | Taiwan (1) |
| Andrews *et al.* 2013 | KC161098 | StLo102 | H 112 | Taiwan (1) |
| Andrews *et al.* 2013 | KC161113 | StLo117 | H 75 | Zanzibar (2) |
| Andrews *et al.* 2013 | KC161114 | StLo118 | H 76 | Zanzibar (2) |
| Andrews *et al.* 2013 | KC161115 | StLo119 | H 77 | Zanzibar (1) |
| Oremus *et al.* 2007 | EF558737 | Slo02FP01 | H 29 | Society Islands: Moorea (7), Tahiti (1) |
| Oremus *et al.* 2007 | EF558738 | Slo02FP02 | H 30 | Society Islands: Moorea (5) |
| Oremus *et al.* 2007 | EF558739 | Slo02FP03 | H 21 | Society Islands: Moorea (1) |
| Oremus *et al.* 2007 | EF558740 | Slo02FP04 | H 31 | Society Islands: Moorea (5), Huahine (1) |
| **Source (Paper)** | **GenBank acession** | **Haplotype code (Original Paper)** | **Haplotype code**  **(This Study)** | **Geographic localization (nº of individuals)** |
| Oremus *et al.* 2007 | EF558741 | Slo02FP05 | H 32 | Society Islands: Moorea (1), Huahine (2), Raiatea-Tahaa (1) |
| Oremus *et al.* 2007 | EF558742 | Slo02FP06 | H 33 | Society Islands: Moorea (2) |
| Oremus *et al.* 2007 | EF558743 | Slo02FP07 | H 34 | Society Islands: Moorea (5), Huahine (2), Bora Bora (2), Raiatea-Tahaa (2) |
| Oremus *et al.* 2007 | EF558744 | Slo02FP08 | H 35 | Society Islands: Moorea (1) |
| Oremus *et al.* 2007 | EF558745 | Slo02FP09 | H 36 | Society Islands: Moorea (3), Bora Bora (1), Raiatea-Tahaa (3) |
| Oremus *et al.* 2007 | EF558746 | Slo02FP11 | H 17 | Society Islands: Moorea (4), Tahiti (8), Huahine (1) |
| Oremus *et al.* 2007 | EF558747 | Slo02FP15 | H 37 | Society Islands: Moorea (7), Bora Bora (1), Raiatea-Tahaa (4) |
| Oremus *et al.* 2007 | EF558748 | Slo02FP20 | H 38 | Society Islands: Moorea (6), Raiatea-Tahaa (1) |
| Oremus *et al.* 2007 | EF558749 | Slo02FP22 | H 39 | Society Islands: Moorea (11) |
| Oremus *et al.* 2007 | EF558750 | Slo02FP27 | H 40 | Society Islands: Moorea (7), Tahiti (24), Bora Bora (1), Raiatea-Tahaa (1) |
| Oremus *et al.* 2007 | EF558751 | Slo02FP36 | H 19 | Society Islands: Moorea (1), Raiatea-Tahaa (1) |
| Oremus *et al.* 2007 | EF558752 | Slo02FP38 | H 14 | Society Islands: Moorea (2) |
| Oremus *et al.* 2007 | EF558753 | Slo02FP45 | H 41 | Society Islands: Moorea (1) |
| Oremus *et al.* 2007 | EF558754 | Slo02FP49 | H 42 | Society Islands: Moorea (1), Raiatea-Tahaa (1) |
| Oremus *et al.* 2007 | EF558755 | Slo03FP18 | H 43 | Society Islands: Bora Bora (1) |
| Oremus *et al.* 2007 | EF558756 | Slo03FP26 | H 44 | Society Islands: Huahine (2) |
| Oremus *et al.* 2007 | EF558757 | Slo03FP32 | H 15 | Society Islands: Huahine (3) |
| Oremus *et al.* 2007 | EF558758 | Slo03FP33 | H 41 | Society Islands: Huahine (1) |
| Oremus *et al.* 2007 | EF558759 | Slo03FP34 | H 45 | Society Islands: Huahine (2), Raiatea-Tahaa (1) |
| Oremus *et al.* 2007 | EF558760 | Slo03FP37 | H 46 | Society Islands: Huahine (2) |
| Oremus *et al.* 2007 | EF558761 | Slo03FP41 | H 47 | Society Islands: Huahine (1), Raiatea-Tahaa (2) |
| Oremus *et al.* 2007 | EF558762 | Slo04FP59 | H 48 | Society Islands: Raiatea-Tahaa (1), Tahiti (1) |
| Oremus *et al.* 2007 | EF558763 | Slo04FP70 | H 49 | Society Islands: Raiatea-Tahaa (1), Nuku Hiva (3) |
| Oremus *et al.* 2007 | EF558764 | Slo04FP78 | H 50 | Society Islands: Nuku Hiva (1) |
| Oremus *et al.* 2007 | EF558765 | Slo04FP79 | H 51 | Society Islands: Nuku Hiva (1) |
| Oremus *et al.* 2007 | EF558766 | Slo04FP82 | H 52 | Society Islands: Nuku Hiva (1) |
| Oremus *et al.* 2007 | EF558767 | Slo04FP83 | H 42 | Society Islands: Nuku Hiva (2) |
| Martien *et al.* 2014 | KY457781 | H1 | H 64 | Guam (1) |
| Martien *et al.* 2014 | KY457782 | H2 | H 53 | 3-Islands (4), Rota (1) |
| Martien *et al.* 2014 | KY457783 | H3 | H 63 | Guam (1) |
| Martien *et al.* 2014 | KY457784 | H4 | H 17 | 3-Islands (9), Rota (2), Guam (4) |
| Martien *et al.* 2014 | KY457785 | H5 | H 17 | 3-Islands (2), Guam (2) |
| Martien *et al.* 2014 | KY457786 | H6 | H 18 | 3-Islands (8), Rota (1) |
| Martien *et al.* 2014 | KY457787 | H7 | H 55 | 3-Islands (1) |
| Martien *et al.* 2014 | KY457788 | H8 | H 15 | 3-Islands (3), Guam (2) |
| Martien *et al.* 2014 | KY457789 | H9 | H 65 | Guam (1) |
| Martien *et al.* 2014 | KY457790 | H10 | H 56 | 3-Islands (8), Guam (1) |
| Martien *et al.* 2014 | KY457791 | H11 | H 12 | 3-Islands (3), Rota (2), Guam (2) |
| Martien *et al.* 2014 | KY457792 | H12 | H 21 | 3-Islands (4), Rota (1), Guam (4) |
| Martien *et al.* 2014 | KY457793 | H13 | H 25 | 3-Islands (1) |
| Martien *et al.* 2014 | KY457794 | H14 | H 61 | Rota (1) |
| Martien *et al.* 2014 | KY457795 | H15 | H 57 | 3-Islands (3), Guam (1) |
| Martien *et al.* 2014 | KY457796 | H16 | H 49 | 3-Islands (3), Rota (2), Guam (4) |
| Martien *et al.* 2014 | KY457797 | H17 | H 42 | 3-Islands (2), Rota (1) |
| Martien *et al.* 2014 | KY457798 | H18 | H 58 | 3-Islands (1) |
| **Source (Paper)** | **GenBank acession** | **Haplotype code (Original Paper)** | **Haplotype code**  **(This Study)** | **Geographic localization (nº of individuals)** |
| Martien *et al.* 2014 | KY457799 | H19 | H 59 | 3-Islands (1) |
| Martien *et al.* 2014 | KY457800 | H20 | H 66 | Guam (1) |
| Martien *et al.* 2014 | KY457801 | H21 | H 67 | Guam (1) |
| Martien *et al.* 2014 | KY457802 | H22 | H 23 | 3-Islands (2) |
| Martien *et al.* 2014 | KY457803 | H23 | H 60 | 3-Islands (1) |
| Martien *et al.* 2014 | KY457804 | H24 | H 20 | Guam (1) |
| Viricel *et al.* 2016 | KX905105 | Hap1 | H 75 | Mayotte (2), Zanzibar (8) |
| Viricel *et al.* 2016 | KX905106 | Hap2 | H 58 | Mayotte (1), Zanzibar (2) |
| Viricel *et al.* 2016 | KX905107 | Hap3 | H 78 | Zanzibar (2) |
| Viricel *et al.* 2016 | KX905108 | Hap4 | H 60 | Zanzibar (1) |
| Viricel *et al.* 2016 | KX905109 | Hap5 | H 8 | Mayotte (1), Zanzibar (1) |
| Viricel *et al.* 2016 | KX905110 | Hap6 | H 79 | Zanzibar (3) |
| Viricel *et al.* 2016 | KX905111 | Hap7 | H 80 | La Reunión (3), Zanzibar (1) |
| Viricel *et al.* 2016 | KX905112 | Hap8 | H 81 | Zanzibar (1) |
| Viricel *et al.* 2016 | KX905113 | Hap9 | H 82 | Zanzibar (1) |
| Viricel *et al.* 2016 | KX905114 | Hap11 | H 83 | Mayotte (2) |
| Viricel *et al.* 2016 | KX905115 | Hap12 | H 84 | Mayotte (1) |
| Viricel *et al.* 2016 | KX905116 | Hap13 | H 85 | Mayotte (3) |
| Viricel *et al.* 2016 | KX905117 | Hap14 | H 74 | Mayotte (1) |
| Viricel *et al.* 2016 | KX905118 | Hap15 | H 86 | Mayotte (1) |
| Viricel *et al.* 2016 | KX905119 | Hap16 | H 87 | Mayotte (1) |
| Viricel *et al.* 2016 | KX905120 | Hap17 | H 75 | Mayotte (1) |
| Viricel *et al.* 2016 | KX905121 | Hap18 | H 76 | Mayotte (2) |
| Viricel *et al.* 2016 | KX905122 | Hap19 | H 88 | Mayotte (1) |
| Viricel *et al.* 2016 | KX905123 | Hap20 | H 89 | Mayotte (1) |
| Viricel *et al.* 2016 | KX905124 | Hap22 | H 89 | Mayotte (1) |
| Viricel *et al.* 2016 | KX905125 | Hap23 | H 90 | La Reunión (4) |
| Viricel *et al.* 2016 | KX905126 | Hap24 | H 91 | La Reunión (1) |
| Viricel *et al.* 2016 | KX905127 | Hap25 | H 92 | La Reunión (3) |
| Viricel *et al.* 2016 | KX905128 | Hap26 | H 93 | La Reunión (1) |
| Viricel *et al.* 2016 | KX905129 | Hap27 | H 94 | La Reunión (1) |
| Viricel *et al.* 2016 | KX905130 | Hap28 | H 95 | La Reunión (1) |
| Viricel *et al.* 2016 | KX905131 | Hap29 | H 26 | La Reunión (1) |
| Viricel *et al.* 2016 | KX905132 | Hap30 | H 1 | La Reunión (1) |
