# Supplementary figures and images for: Low mtDNA diversity in a highly differentiated population of spinner dolphins (Stenella longirostris) from the Fernando de Noronha Archipelago, Brazil
Source: PLoS One. 2020 Apr 7;15(4):e0230660. doi: 10.1371/journal.pone.0230660 (PMC7138316; doi:10.1371/journal.pone.0230660)

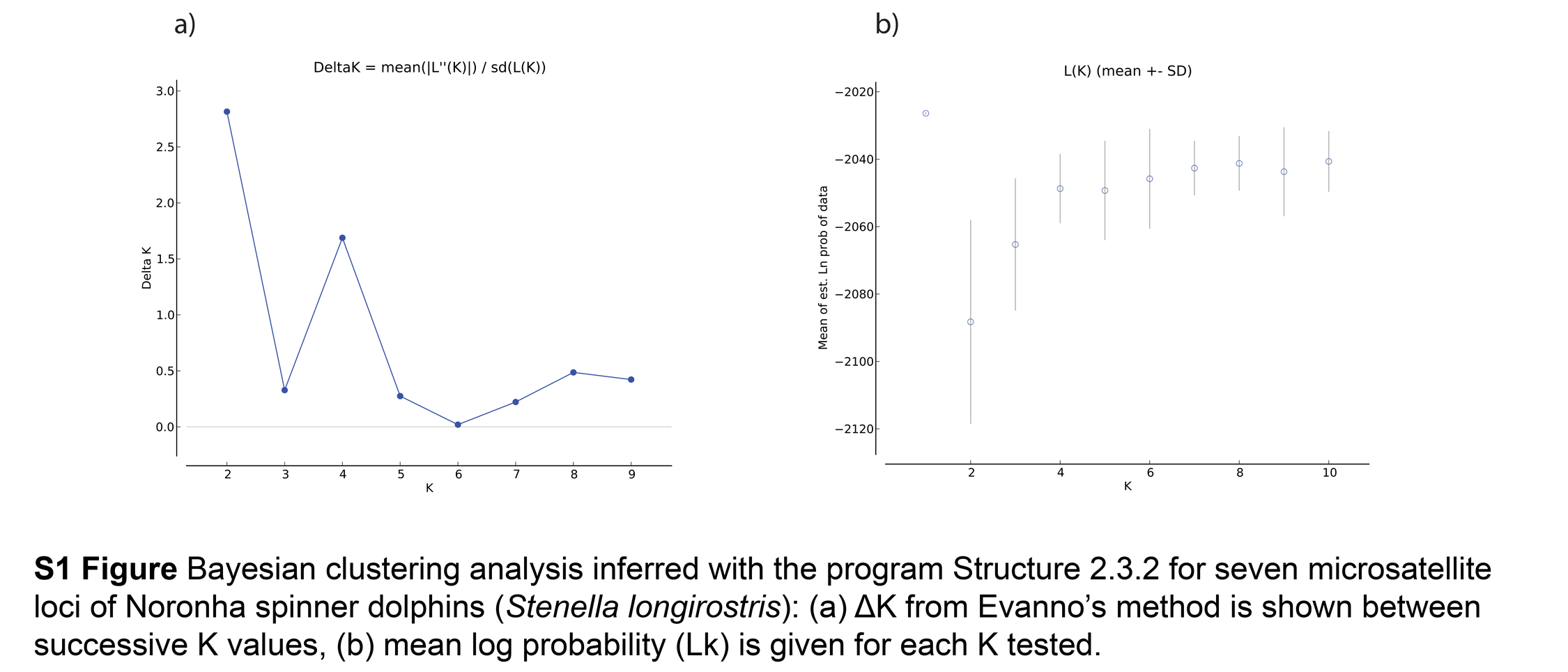

Supplement: S1 Fig — Bayesian clustering analysis inferred with the program Structure 2.3.2 for seven microsatellite loci of Noronha spinner dolphins (Stenella longirostris): (a) ΔK from Evanno’s method is shown between successive K values, (b) mean log probability (Lk) is given for each K tested. (TIF) [file pone.0230660.s002.tif]

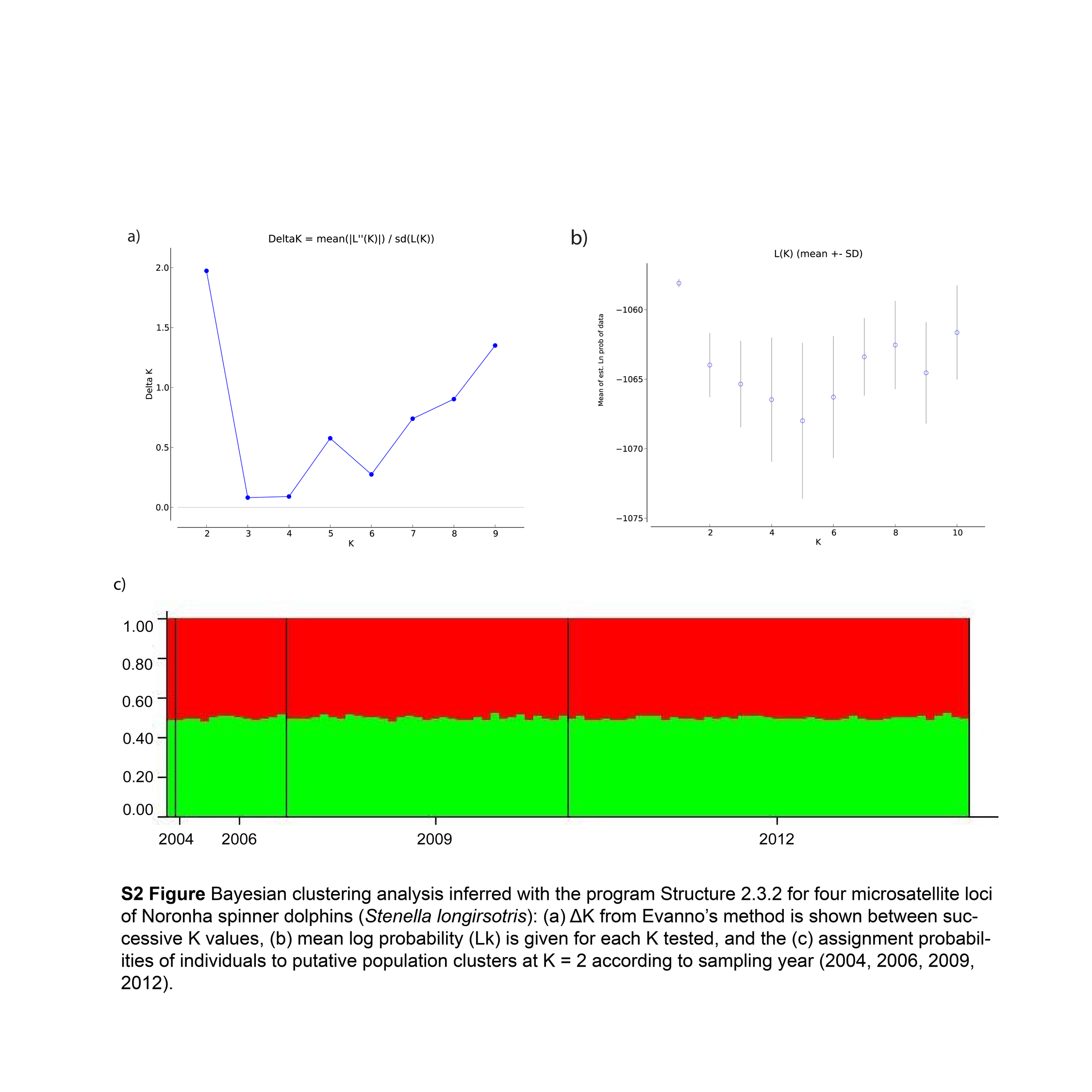

Supplement: S2 Fig — Bayesian clustering analysis inferred with the program Structure 2.3.2 for four microsatellite loci of Noronha spinner dolphins (Stenella longirsotris): (a) ΔK from Evanno’s method is shown between successive K values, (b) mean log probability (Lk) is given for each K tested, and the (c) assignment probabilities of individuals to putative population clusters at K = 2 according to sampling year (2004, 2006, 2009, 2012). (TIF) [file pone.0230660.s003.tif]

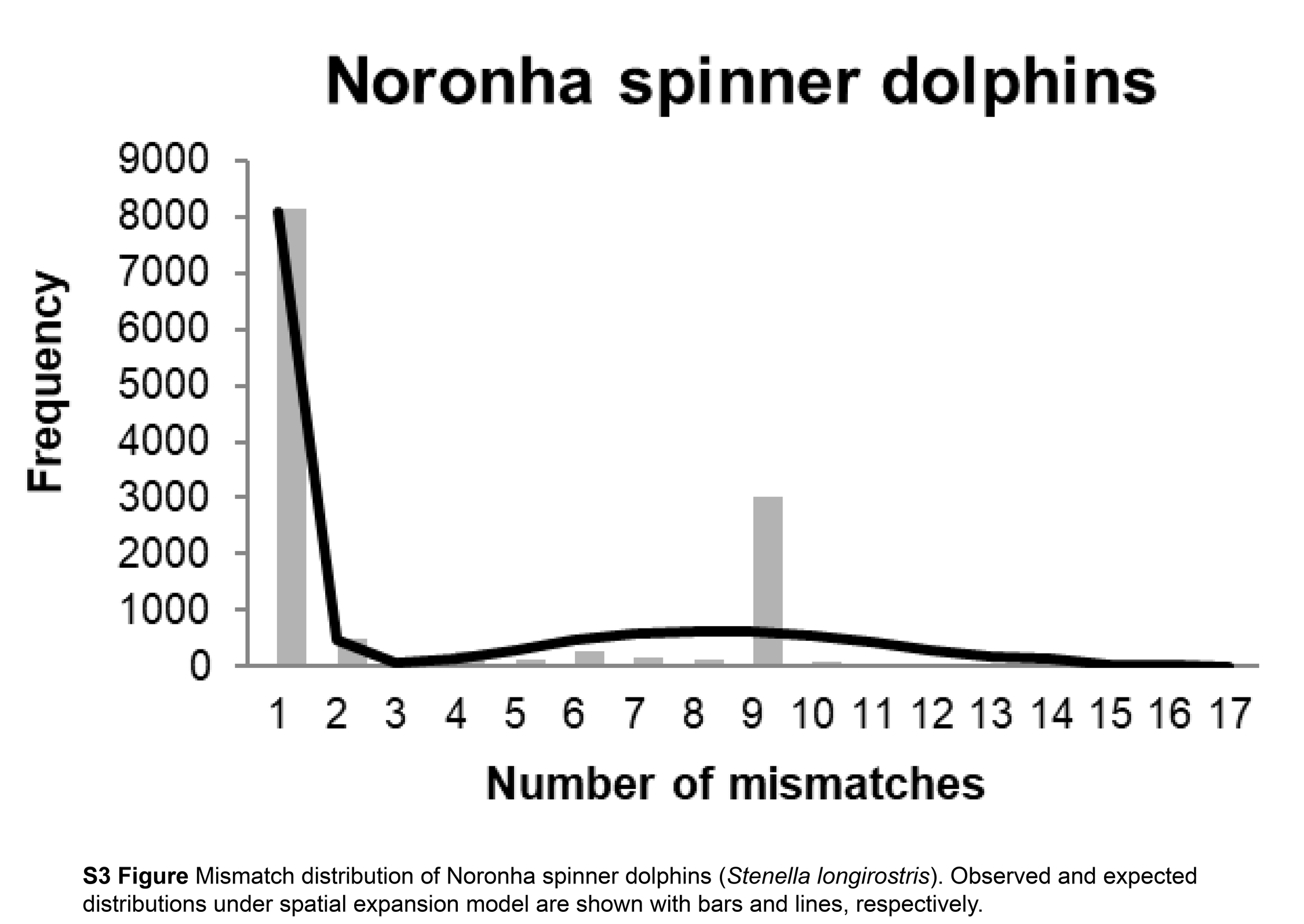

Supplement: S3 Fig — Observed and expected distributions under spatial expansion model are shown with bars and lines, respectively. (TIF) [file pone.0230660.s004.tif]
